# Application of Spectrophotometric Fingerprint in Cluster Analysis for Starch Origin Determination

**DOI:** 10.17113/ftb.58.01.20.6239

**Published:** 2020-03

**Authors:** Nikola Sakač, Maja Karnaš, Jasminka Dobša, Marija Jozanović, Vlatka Gvozdić, Elvira Kovač-Andrić, Marija Kraševac Sakač, Bojan Šarkanj

**Affiliations:** 1 University of Zagreb, Faculty of Geotechnical Engineering, Hallerova 7, HR-42000 Varaždin, Croatia; 2 Josip Juraj Strossmayer University of Osijek, Faculty of Agriculture, Vladimira Preloga 1, 31000 Osijek, Croatia; 3 University of Zagreb, Faculty of Organization and Informatics, Pavlinska 2, 42000 Varaždin, Croatia; 4 Josip Juraj Strossmayer University of Osijek, Department of Chemistry, Cara Hadrijana 8/A, 31000 Osijek, Croatia; 5 University North, Dr Žarko Dolinar Square 1, 43000 Koprivnica, Croatia

**Keywords:** starch botanical origin, starch-triiodide complex, cluster analysis

## Abstract

The botanical origin of starch is of importance in industrial applications and food processing because it may influence the properties of the final product. Current microscopic methods are time-consuming. Starch consists of an origin-dependent amylose/amylopectin ratio. Triiodide ions bind characteristically to the amylose and amylopectin depending on the botanical origin of the starch. The absorbance of the starch-triiodide complex was measured for: wheat, potato, corn, rye, barley, rice, tapioca and unknown origin starch; and within the different cultivars. Each starch sample had specific parameters: starch-triiodide complex peak wavelength maximum (*λ*_max_/nm), maximum absorbance change at *λ*_max_ (Δ*A*) and *λ*_max_ shift towards the unknown origin starch sample values. The visible absorption spectra (500-800 nm) for each starch sample were used as a unique fingerprint, and then elaborated by cluster analysis. The cluster analysis managed to distinguish data of two clusters, a cereal type cluster and a potato/tapioca/rice starch cluster. The cereal subclusters extensively distinguished wheat/barley/rye starches from corn starches. Data for cultivars were mostly in good agreement within the same subclaster. The proposed method that combines cluster analysis and visible absorbance data for starch-triiodide complex was able to distinguish starch of different botanical origins and cultivars within the same species. This method is simpler and more convenient than standard time-consuming methods.

## INTRODUCTION

Starch is one of the major natural polysaccharides. It is widely used in numerous branches of industry, *e.g*. food, paper, adhesive, textile, cosmetic and biorefinery (*1*-*4*). One of the most important factors that dictate starch processing and final product quality is its botanical origin (*5*); confirming the need for a fast and reliable method for starch origin determination. Determination of starch origin in artificial food is also one of current issues.

Starch is found in nature as granules, an immense and highly organized structure. Botanical origin of starch dictates the ratio and the way amylose and amylopectin are associated and packed (*6*-*8*). The branched molecules of amylose and amylopectin from various origins have their own characteristic structures such as molecular size, inner chain length, and the number of side chains. For example, wheat amylose probably contains only a small number of very large branched molecules, whereas sweet potato amylose has a small number of relatively large unbranched molecules (*9*).

Many physicochemical properties of amylose and amylopectin (*10*, *11*), such as iodine binding capacity and degree of polymerisation (DP), depend on the botanical origin of the starch. The amylose content and the amylose-amylopectin ratio have traditionally been measured by iodine-binding methods (*12*) using various techniques (*13*, *14*), especially spectroscopic (*15*). These methods are based on the capacity of amylose and amylopectin to form blue coloured helical inclusion complexes with iodine. Different spectroscopic (visible light absorption) characteristics of the starch–triiodide complex vary with chain length, which is dependent on the botanical origin of starch (*16*). It is also known that plant species within a variety have similar amylose/amylopectin ratio (within certain deviations) and the knowledge of their spectroscopic properties could contribute to starch origin identification (*17*, *18*).

The typical origin analysis of starch includes indirect techniques that measure the differences in the physical and chemical properties of the starch (*19*). The starch origin identification is based on enzymatic hydrolysis of starch granules (*20*, *21*), use of SDS-PAGE for starch granule-associated protein (SGAP) determination (*5*), and the use of peptide mass fingerprinting of granule-bound starch synthase (*22*). These methods are time consuming and require specific instrumentation. Therefore, the main goal of our research is to develop a fast and inexpensive method for identification of starch origin.

The method described in our previous work (*23*) determines starch origin based on direct potentiometric measurements of starch-triiodide complexes and the use of principal components data analysis (PCA). That method could distinguish between the starch types of different botanical origins based on their chemical differences, amylose/amylopectin ratios and specific triiodide ion binding affinities to amylose and amylopectin.

In this paper a new method is proposed that measures spectrophotometric, instead of potentiometric properties of the starch-triiodide complex. To speed up and simplify the determination, the botanical origin-specific triiodide ion binding affinities to amylose and amylopectin were used, instead of time-consuming determination of amylose and amylopectin content and iodine-binding capacity of each sample. The ability of the proposed method to distinguish starch types of different botanical origins is verified by statistical data analysis using hierarchical clustering of the complete visible spectral data of the starch-triiodide complex. The method is particularly suitable for use in laboratories equipped with simple UV-Vis spectrophotometers.

## MATERIALS AND METHODS

### Samples and reagents

Starch samples were isolated from wheat (Srpanjka and Golubica), corn, rye (Eho, Danovski and Conduct), barley (Barun, Vanessa), rice and tapioca, which were obtained at the local market store in Croatia. Waxy corn, wheat and potato commercial starch samples were purchased from Sigma-Aldrich, Merck (Darmstadt, Germany). Commercial starch of unknown (US) origin was purchased from Kemika (Zagreb, Croatia). Potassium triiodide solution was prepared using iodine and potassium iodide, both purchased from Sigma–Aldrich, Merck. Glacial acetic acid was purchased from Panreac (Barcelona, Spain) and sodium acetate trihydrate was purchased from J.T. Baker (Deventer, the Netherlands).

### Starch sample preparation

The sample seed coats were peeled off, and an alkaline steeping method (*24*, *25*) was used to separate the starch. The preparation steps included pH adjustment, blending at low speed (blender model MMBH4P3W, 1600 W; BOSCH, Dortmund, Germany), filtrations, three steps of resuspension in deionised water, drying (dryer model ST-05; Instrumentaria, Zagreb, Croatia), grinding with a mortar and pestle to pass through 60-mesh sieve and dry storage.

### Starch solution preparation

The starch was dried for 90 min at 130 °C by spreading approx. 2 g of air-dried soluble starch in a thin layer over the bottom of a weighing bottle with a lid. The starch solution was prepared by dissolving the amount of starch equivalent to 0.2 g of anhydrous starch (concentration 2 g/L) in a previously prepared acetate buffer solution (pH=6.0) in a closed 100-mL volumetric flask. After the solution was heated and stirred for 10 min in a sonic bath (RK 514 BH; Bandelin Sonorex, Berlin, Germany), it was allowed to cool to room temperature and was then diluted to 100 mL with deionized water in a volumetric flask. For each starch type, five independent solutions were prepared each day to avoid microbial degradation.

### Potassium triiodide solution preparation

The potassium triiodide solution was prepared by dissolving solid iodine (600 µM) in a 0.03 M potassium iodide solution. Iodine is very toxic and easily sublimates, which makes it difficult to weigh; the weighing procedure should be fast but accurate. Iodine has low water solubility; thus, it was dissolved in the following order. First, potassium iodide was dissolved in a small volume of water, making it possible to dissolve slowly the iodine. After the iodide solution was vigorously stirred, the iodine was completely dissolved, and the volumetric flask was filled to the mark.

### Sepctrophotometric measurements

The spectrophotometric measurements were performed on an Avantes optical system: AvaSpec-ULS3648 high resolution spectrophotometer with *d*=400 µm optical fibre, measuring cuvette holder and AvaLight-DH-S light source, with AvaSoft v. 7.0. software (all from Avantes, Apeldoorn, the Netherlands) (*26*). Sonic bath (RK 514 BH; Bandelin Sonorex) was used for sample solution preparations.

For spectrophotometric measurements, five independent series of starch triiodide solutions were prepared. First, six 50-mL volumetric flasks were incrementally filled with *V*(starch)=0, 0.25, 0.50, 0.75, 1.0 and 1.25 mL. The previously prepared potassium triiodide solution was then added to the starch-filled volumetric flasks in opposite incremental volume order *V*(I_3_^-^)=1.25, 1.0, 0.75, 0.5, 0.25 and 0 mL. The volumetric flasks were filled to the mark with deionized water, stirred in a sonic bath for 5 min and then were ready for further investigation. Subsequently, the spectrophotometric measurement data were collected and analysed using licensed Statistica v. 12 software (*27*).

## RESULTS AND DISCUSSION

### Spectrophotometric characterization of starch-triiodide complex

**Fig. 1:**
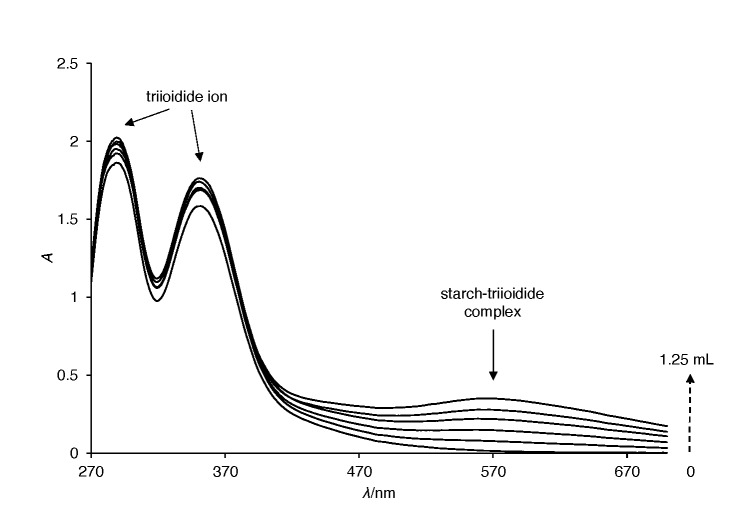
Absorption spectra of commercial starch of unknown origin at *V*=0, 0.25, 0.50, 0.75, 1.0 and 1.25 mL

Starch samples were characterized by measuring starch triiodide complex absorption spectra. Fig. 1 shows the spectra of the commercial starch of unknown origin that was used for comparison with other starch types.

The spectra exhibited three different peaks in the recorded UV-Vis region. The first one, with an absorption maximum at 564 nm, corresponded to a starch-triiodide complex. The other two, with maxima at 342 and 285 nm, were assigned to triiodide/iodide ions.

With an increase of starch concentration, the starch-triiodide complex concentration also increased, resulting in absorbance enhancement at the starch-triiodide wavelength maximum. At the same time, the absorbance of the other two peaks decreased due to the binding of triiodide to starch.

**Fig. 2:**
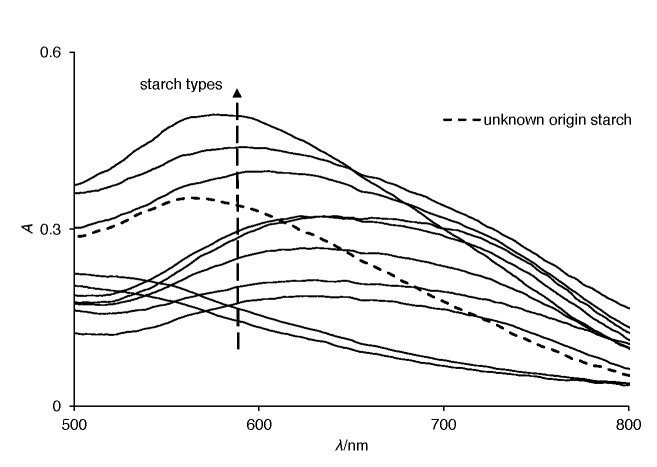
Absorption spectra for starch samples of different botanical origins. The arrow represents a vertical separation (at a given wavelength) for a more straightforward presentation of starch absorption lines. From bottom: waxy corn, corn, wheat (Srpanjka), rye (Conduct), wheat, barley (Vanessa), rye (Eho), rye (Danovski), barley (Barun), wheat (Golubica), commercial starch of unknown origin, tapioca, rice and potato

Because the amylose-amylopectin ratio in starch and specific triiodide ion binding affinities to amylose and amylopectin depend directly on botanical starch origin, there is a considerable difference in the starch-triiodide spectra for different starch types (Fig. 2).

The difference is noticeable in the wavelength area of the starch-triiodide complex where peaks vary in their heights and maximum wavelength values. This was supported by the literature data (*14*), where the concentration of triiodide ion is consumed by the inner cavities of helical structures of amylose and amylopectin. The inclusion complex of amylose with triiodide exhibits the absorption spectra with the maximum peaks at wavelengths higher than 620 nm. On the other hand, amylopectin inclusion complex with triiodide ion exhibits the adsorption spectra with the maximum peaks at lower wavelengths, approx. 540 nm, and interferes with the amylose determination with colorimetric methods (*12*, *13*).

The parameters for starch-triiodide complex in Table 1 present a maximum peak wavelength and a change of absorbance value at maximum peak wavelength for the same measuring condition for each starch sample towards the value of a maximum peak wavelength shift and the maximum peak wavelength of the unknown starch sample. These values were used for rough differentiation of starch type before the statistical analysis (Table 1).

**Table 1:** Measured parameters from the absorbance spectra for all starch types. The commercial starch of unknown origin was used as a zero for comparison

Starch origin (cultivar)	*λ*_max_*/nm	∆*Ā*	∆*λ*/nm
Unknown	564	0.337	0
Potato	571	0.474	+7
Corn	510	0.221	-46
Waxy corn	520	0.155	-44
Tapioca	600	0.398	+36
Rye (Eho)	630	0.310	+66
Rye (Danovski)	635	0.318	+71
Rye (Conduct)	623	0,255	+59
Wheat (Golubica)	633	0.312	+69
Wheat (Srpanjka)	630	0.197	+66
Wheat	632	0.270	+68
Barley (Barun)	632	0.326	+68
Barley (Vanessa)	623	0.246	+59
Rice	590	0.422	+26

*Average values of 5 measurements

at *λ*_max_ and *V*(starch increment)=1.25mL

The commercial starch of unknown origin was used as a zero for *λ*_max_ comparison to other starch types (presented as ∆*λ*). Rye (Danovski cv.) showed the highest positive shift (+71 nm), waxy corn and corn starch the highest negative (-44 and -46) and potato starch (+7) the lowest shift towards the commercial starch of unknown origin. Rye, barley and wheat varieties exhibited *λ*_max_ in very narrow *λ* region. The absorbance increase (∆*A*) at each *λ*_max_ was calculated. Potato and rice starch showed the highest absorbance increase, 0.474 and 0.422, respectively. This indicates that the highest amount of triiodide is complexed with starch.

In this paper we used the usually negative influence of amylopectin (that immobilizes some triiodide ions) on amylose determination (false positive result) as an advantage to get the whole absorbance spectra and actually get the individual fingerprint of each starch sample. These fingerprint datasets were then analysed for the first time and compared with the cluster analysis.

### Statistical data evaluation using cluster analysis

Clustering aims at discovering natural groupings of items, thus revealing interesting data patterns in their relations (*28*, *29*). A matrix of similarities between the items is used to define their groups. In this research we use the most common Euclidean distance between the items as a measure of similarity. Clustering methods are divided in two groups: hierarchical clustering and non-hierarchical clustering. Furthermore, hierarchical methods can be agglomerative or divisive. Clustering by agglomerative methods starts with the individual item and groups the most similar items until all objects are contained in the same cluster, while divisive hierarchical methods work in the opposite direction: an initial single group of items is divided into two subgroups so that the items in two groups are dissimilar. The result of hierarchical clustering is displayed in a form of a diagram called dendrogram. The way of merging clusters together in agglomerative hierarchical method is determined by the linkage. The most common methods are single linkage (groups are merged according to the distance between their nearest members of groups), complete linkage (groups are merged according to the distance between their farthest members) and average distance (groups are merged according to the average distance between their members). In this paper we used agglomerative hierarchical method with complete linkage. The advantage of complete linkage is that it is efficient in discerning poorly separated clusters.

In addition to visual inspections of starch spectra and a rough differentiation before the statistical analysis of starch type (Table 1), we examined the spectra by the cluster analysis. Although we used complete visible spectra of the starch-triiodide complex (fingerprints) for all starch increments, only the data for starch volume increments of 1.25 mL allowed starch samples to be distinguished using cluster analysis, and these concentration data were used for further evaluation.

**Fig. 3:**
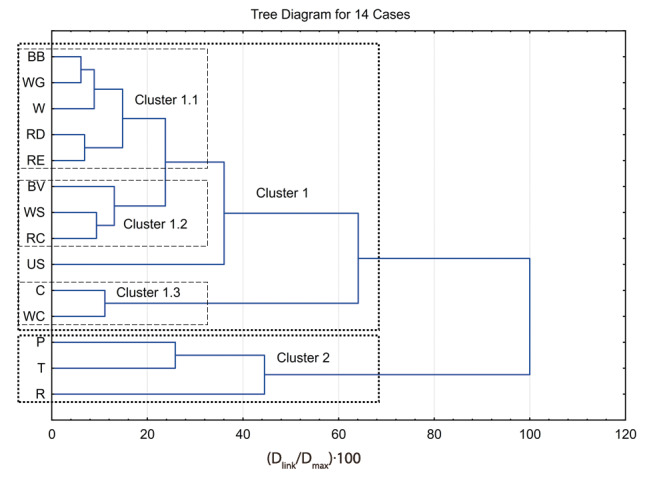
A dendrogram of hierarchical clustering of centroids for starch samples belonging to the same source. Linkage distance is shown relatively according to maximal linkage distance between the groups. Different origins of starch samples are: BB=barley Barun, WG=wheat Golubica, W=wheat, RD=rye Danovski, RE=rye Eho, BV=barley Vanessa, WS=wheat Srpanjka, RC=rye Conduct, US=commercial starch of unknown origin, C=corn, WC=waxy corn, P=potato, T=tapioca, R=rice

Fig. 3 shows a dendrogram of hierarchical clustering of average values (centroids) of starch samples. Here we can observe that starch samples are divided in two main clusters (cluster 1 and cluster 2). The first cluster consists of three smaller subclusters: first two (clusters 1.1 and 1.2) contain cereals (wheat, rye and barley) with model starch (US) attached to these two clusters, while the third subcluster (cluster 1.3) contains corn starch samples. The second cluster contains potato, tapioca and rice. Sources from the second cluster seem to be well separated from others.

The unknown starch (US) sample was placed within cluster 1, but it was separated from clusters 1.1 and 1.2, and also distinguished from cluster 1.3. The reason for this could be explained through the origin of the unknown origin starch. At our request, the producer provided the information that this starch (US) was of wheat origin but slightly debranched by the enzymes. When observing the position of the US in cluster 1, this makes sense, it is close enough to cereal-type starch samples in clusters 1.1 and 1.2 and still quite distinguished from corn-type starch in cluster 1.3.

**Fig. 4:**
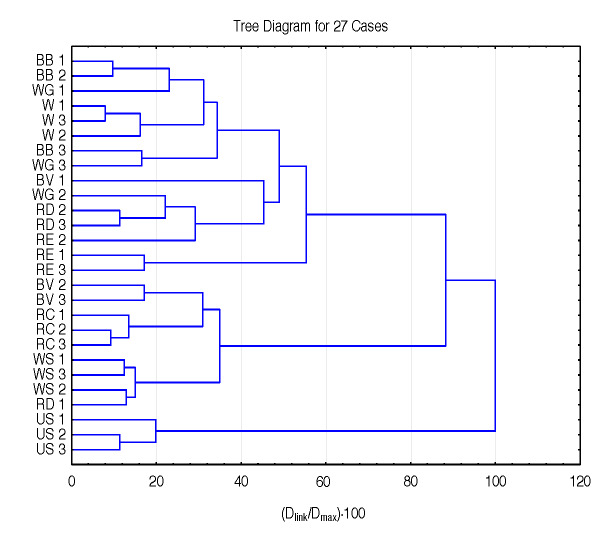
A dendrogram of hierarchical clustering of samples belonging to sources in the first two subclusters of the first cluster on dendrogram shown on Fig. 3 (subclusters 1.1 and 1.2). Samples of commercial starch of unknown origin are attached to these subclusters. Abbreviations: BB=barley Barun, WG=wheat Golubica, W=wheat, BV=barley Vanessa, RD=rye Danovski, RE=rye Eho, RC=rye Conduct, WS=wheat Srpanjka, US=commercial starch of unknown origin

Fig. 4 shows dendrogram of hierarchical clustering of samples belonging to starch samples from the first two subclusters (clusters 1.1 and 1.2) of the first cluster in the dendrogram in Fig. 3 with the source of model starch (US) which is grouped into these subclusters. Each source is presented by three samples. Generally speaking, it can be observed that samples from the same species (origin) but different cultivars are mainly well grouped, which confirms our hypothesis that the proposed method, using the visible spectra (depending on amylose/amylopectin ratio) as a starch fingerprint and their evaluation with cluster analysis, is capable of distinguishing starch samples based on their origin but also on their cultivars. As explained above, the position of US starch is distanced and makes a separate subclaster, but it is still positioned in the cereal-type cluster 1 due to its natural origin.

**Fig. 5:**
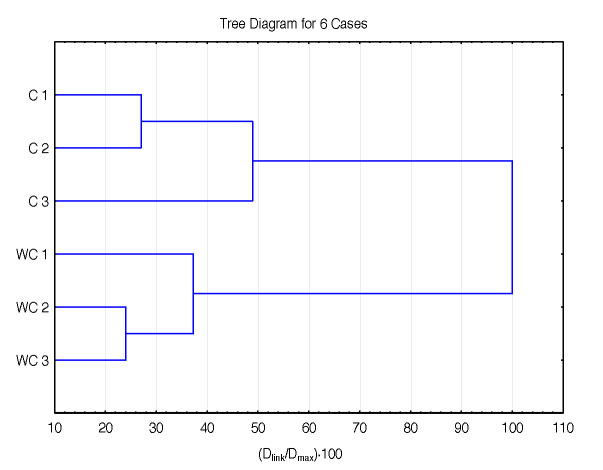
A dendrogram of hierarchical clustering of samples belonging to sources in the second subcluster of the first cluster in Fig. 3 (cluster 1.3). Abbreviations: C=corn, WC=waxy corn

Fig. 5 shows dendrogram of hierarchical clustering of samples of sources in the third subcluster of the first cluster in Fig. 3 (corn and waxy corn). The corn samples were distinguished due to their unique visible spectral fingerprint, which is shifted to lower wavelengths because of their high amylopectin content. But it is interesting to observe the position of corn subcluster 1.3, which is still within the cereal-type cluster 1.

**Fig. 6:**
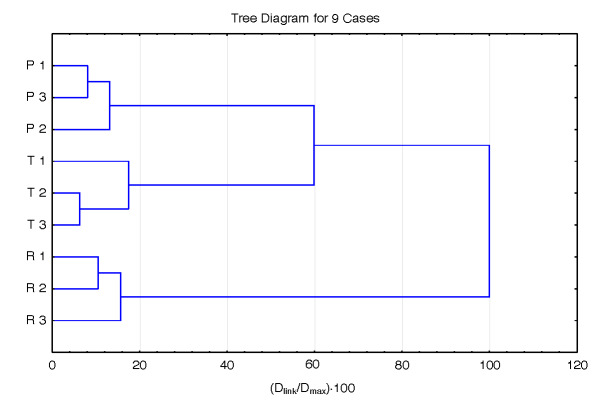
A dendrogram of hierarchical clustering of samples belonging to sources in the second cluster of dendrogram in Fig. 3 (cluster 2). Abbreviations: P=potato, T=tapioca, R=rice

On the contrary, Fig. 6 shows that hierarchical clusters of samples of sources in the second cluster in Fig. 3 (potato, tapioca and rice) are totally different and separated in their own cluster based on their unique visible spectral fingerprint. Tapioca and potato have indeed different properties compared to cereal-type starch, and rice starch granules are the smallest known to exist in cereal grains (*30*). This shows that the proposed method is reliable in recognizing starch samples from the investigated sources.

The observed distances in different starch spectra separated in clusters (see Fig. 2) were in agreement with our assumption that the visible absorbance spectral fingerprint is a result of an origin-dependent amylose-amylopectin ratio (*17*, *18*) and specific triiodide binding to different starch types (*9*), also shown in Table 2 (*9**,**14**,**31*). Thus, specific differences in the visible absorbance spectra of starch-triiodide complex (fingerprint) could be exploited to distinguish starch samples based on their botanical origins (Fig. 3). It should be noted that among all starch samples, the absorption spectra of potato, tapioca and rice are remarkably different. These differences can be explained by the different amylose–amylopectin ratios, degrees of polymerization, different helical structures, granule size and other physical and chemical properties of the starch. The presented method offers a potential application in artificial food determination and also in food processing industry for starch origin control and increase in the quality of the final product.

**Table 2:** Iodine affinity, amylose and amylopectin content in starch from various origins (adapted from (*9*, *14*, *31*))

Starch origin	Iodine affinity	Amylose content/%	Amylopectin content/%
Rice	4	17.5	82.5
Wheat	4.86	21.7	78.3
Barley	6.08	27.5	72.5
Corn	5.18	21.5	78.5
Potato	4.44	21	79
Sweet potato	4.18	20.7	79.3
Chestnut	4.32	21.6	78.4
Sago	5.16	25.8	74.2
Rye	N.A.	31.1	68.9
Tapioca	N.A.	19.7	80.3

N.A.=not available

## CONCLUSION

The presented research successfully combined spectrophotometric data obtained by measuring the starch-triiodide complex (as a unique fingerprint of each starch sample) and statistical cluster analysis to distinguish the starch samples based on different origin, including among different cultivars. The proposed method uses simple instrumentation, is more convenient and easier to use than the standard microscopic methods, and provides an alternative to our recently developed electrochemical methods. The future work will include more comprehensive research on testing the performance of the proposed method. The method will be tested by supervised machine learning method of automatic classification of samples data and compared to other methods for recognition of starch source. The presented method offers a potential application in artificial food determination and also in food processing industry for starch origin control and increase in quality of final product, but potentially, also in botanic determinations and origin relations of different species.
